# Phosphorylation Dynamics of JNK Signaling: Effects of Dual-Specificity Phosphatases (DUSPs) on the JNK Pathway

**DOI:** 10.3390/ijms20246157

**Published:** 2019-12-06

**Authors:** Jain Ha, Eunjeong Kang, Jihye Seo, Sayeon Cho

**Affiliations:** Laboratory of Molecular and Pharmacological Cell Biology, College of Pharmacy, Chung-Ang University, Seoul 06974, Korea; joehalee@gmail.com (J.H.); ejaykang@gmail.com (E.K.); seojh0228@gmail.com (J.S.)

**Keywords:** mitogen-activated protein kinase pathway, c-Jun N-terminal kinase pathway, dual-specificity phosphatase, dephosphorylation

## Abstract

Protein phosphorylation affects conformational change, interaction, catalytic activity, and subcellular localization of proteins. Because the post-modification of proteins regulates diverse cellular signaling pathways, the precise control of phosphorylation states is essential for maintaining cellular homeostasis. Kinases function as phosphorylating enzymes, and phosphatases dephosphorylate their target substrates, typically in a much shorter time. The c-Jun N-terminal kinase (JNK) signaling pathway, a mitogen-activated protein kinase pathway, is regulated by a cascade of kinases and in turn regulates other physiological processes, such as cell differentiation, apoptosis, neuronal functions, and embryonic development. However, the activation of the JNK pathway is also implicated in human pathologies such as cancer, neurodegenerative diseases, and inflammatory diseases. Therefore, the proper balance between activation and inactivation of the JNK pathway needs to be tightly regulated. Dual specificity phosphatases (DUSPs) regulate the magnitude and duration of signal transduction of the JNK pathway by dephosphorylating their substrates. In this review, we will discuss the dynamics of phosphorylation/dephosphorylation, the mechanism of JNK pathway regulation by DUSPs, and the new possibilities of targeting DUSPs in JNK-related diseases elucidated in recent studies.

## 1. Phosphorylation-Dephosphorylation: The Scope of Thermodynamics

**Figure 1:**
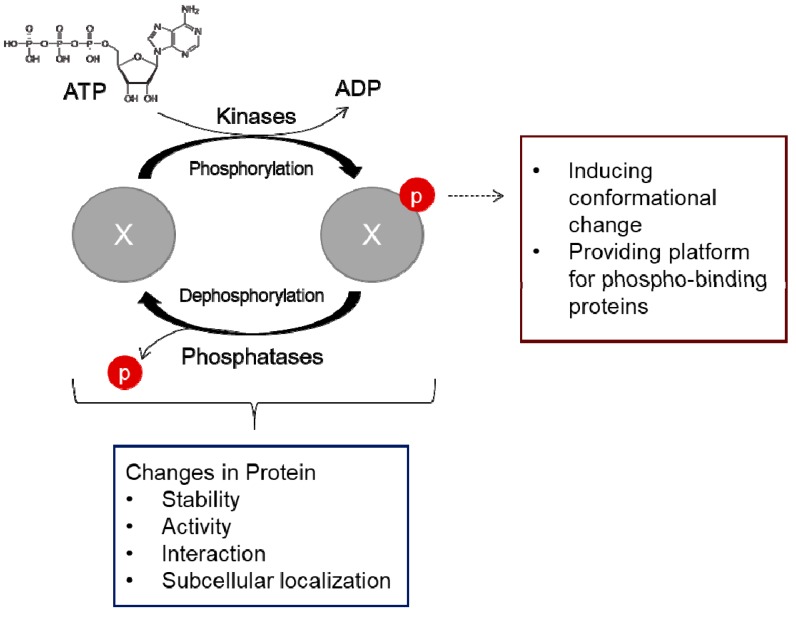
Reversible phosphorylation by kinase and phosphatase. Phosphorylation is an essential post-translational modification that is mediated by kinases. Reversible phosphorylation induces conformational change within the protein or provides a platform for phospho-binding proteins, which in turn triggers alterations in protein stability, activity, interaction, or subcellular localization. Because phosphorylation regulates diverse protein functions, it should be tightly controlled by the reverse reaction—dephosphorylation catalyzed by phosphatases. X represents a protein that is reversibly phosphorylated and dephosphorylated, and p stands for a phosphate. ATP, adenosine triphosphate; ADP, adenosine diphosphate.

Cellular regulatory mechanisms respond specifically and robustly to extracellular stimuli. Post-translational modification (PTM) indicates covalent modifications of proteins after translation, such as protein methylation, glycosylation, acetylation, sumoylation, and ubiquitination. PTM plays a vital role in the control of protein activity, stability, and subcellular localization, thereby contributing to intracellular regulation. Among these modifications, protein phosphorylation is one of the most studied PTMs. Based on phosphoproteomics approaches, phosphorylation at serine was reported to be most abundant (86.4%), followed by threonine (11.8%), and tyrosine (1.8%) in HeLa cells [1]. In addition, more than 30% of eukaryotic proteins were observed to be phosphorylated [1]. As phosphorylation alters protein function, the reversible regulation of phosphorylation is essential for maintaining cellular physiology within a normal range (Figure 1) [2,3].

**Figure 2:**
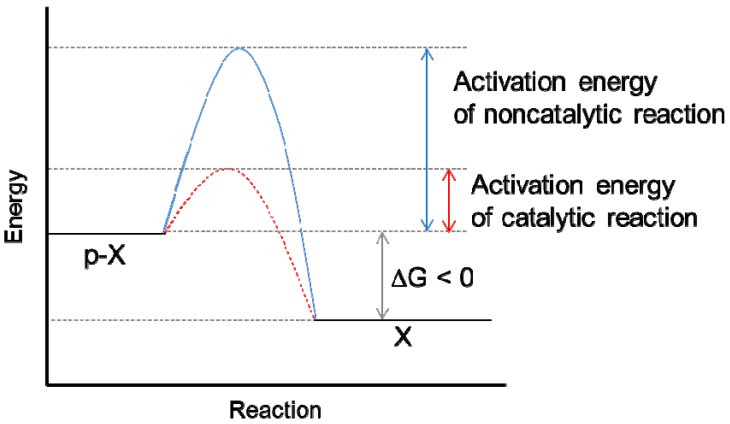
Dephosphorylation reaction with or without phosphatase. Because a phosphorylated substrate (p-X) is at a higher free energy than the unphosphorylated form (X), it is thermodynamically prone to lose a phosphate eventually. However, for the p-X to lose its phosphate and become X, energy is required. Due to this high-energy barrier (indicated as a blue line), non-catalytic reactions take a long time. With a catalytic enzyme (in this case, a phosphatase), the activation energy (indicated as a red dashed line) of the enzymatic reaction is greatly reduced compared to that of a non-catalytic reaction.

To further understand the balance between phosphorylation and dephosphorylation, the thermodynamics of Gibbs free energy will be briefly discussed. A change in Gibbs free energy (∆G), an indicator of the direction of chemical reactions, is dependent on the temperature and molar ratio of the reactants and products. A negative value of ∆G means that the reaction proceeds spontaneously [4]. Most phosphodiester hydrolysis reactions have negative ∆G values, which means that the dissociation of phosphate is prone to occur [5]. However, “spontaneous” dephosphorylation is challenging to identify in biological systems even though it has a negative net change in energy because it proceeds at a slow rate due to a high activation energy (Figure 2). Acid hydrolysis of phosphoamino acids in 1 N HCl results in approximately 40% of phosphoserine and 60% of phosphothreonine remaining after 24 h [6,7], indicating that non-enzymatic hydrolysis of phosphoproteins in neutral solutions would require a much longer time. When a protein phosphatase is present, it dramatically shortens the reaction time by lowering the activation energy. The catalytic activity of all other enzymes, including phosphatases, arises from this ability to lower the activation energy of the reaction. In the case of catalysis by vaccinia H1-related (VHR) dual-specificity phosphatase, the calculated energy barrier was 16.4 kcal/mol, which was less than half the energy barrier of non-catalytic hydrolysis [8]. The change in activation energy barrier affects the reaction rate [9]. Denu et al. reported that the turnover of *p*-nitrophenyl phosphate (pNPP) by wild-type (WT) VHR phosphatase was more than 6000-fold of that of the VHR S131A/D92N inactive mutant [9]. In addition, a simulation study by Kolmodin and Aqvist suggested that hydrolysis of the phosphoenzyme intermediate by low-molecular-weight protein tyrosine phosphatase (LM-PTP) lowered the activation energy by approximately ~15 kcal/mol compared to non-enzymatic hydrolysis [10].

Regarding enzyme kinetics, *k_cat_* and *K_M_* provide useful information; *k_cat_* indicates the number of substrate molecules catalyzed by an enzyme per second. *K_M_* equals the concentration of a substrate when the reaction velocity is 1/2 of the maximum velocity, and *k_cat_*/*K_M_* equals the enzymatic efficiency. For example, dephosphorylation of tris-phosphorylated insulin receptor peptide by protein tyrosine phosphatase 1B (PTP1B) has a *k_cat_* value of 11.3 ± 0.82 (s^−1^) and a *k_cat_*/*K_M_* value of 1514 (s^−1^ M^−1^), which indicates a highly specific dephosphorylation reaction [11]. To dynamically regulate the cellular signaling and respond to extracellular stimuli, most dephosphorylation of phosphorylated proteins within cells should be catalyzed by protein phosphatases.

To illustrate the roles of phosphatases in signaling networks, we focus on the c-Jun N-terminal kinase (JNK) pathway, and roles of JNK-specific phosphatases, dual-specificity phosphatases (DUSPs) in particular, in this review.

## 2. The c-Jun N-terminal Kinase (JNK) Pathway

Evolutionally conserved mitogen-activated protein kinase (MAPK) pathways are composed of extracellular signal-regulated protein kinase (ERK), p38, and JNK pathways. MAPK pathways are activated by various extracellular factors such as growth factors, pro-inflammatory cytokines, or environmental stresses [12]. These stimuli trigger activation of the MAPK pathway via binding to the membrane receptors, including receptor tyrosine kinases, G-protein-coupled receptor (GPCR), serine/threonine kinase receptors, and inflammatory cytokine receptors [13,14,15,16]. In general, the MAPK pathway is comprised of “three-tiers”: MAPK kinase kinases (MAP3Ks), MAPK kinases (MAP2Ks), and MAPKs. MAP3Ks, serine/threonine kinases in the upper tier, are typically phosphorylated and activated by interactions with small GTP-binding proteins. In turn, activated MAP3Ks phosphorylate and activate MAP2Ks. MAP2Ks then phosphorylate both serine/threonine and tyrosine residues, known as a Thr-E/P/G-Tyr motif on MAPKs, which indicates glutamate (E), proline (P), and glycine (G) in ERK, JNK, and p38 proteins, respectively [17]. MAPKs target downstream substrates, primarily transcription factors. Hence, MAPKs participate in the regulation of gene expression, mitosis, proliferation, cell survival, and apoptosis. As we focus on the regulation of JNK pathway in this review, JNK will be discussed in depth.

**Figure 3:**
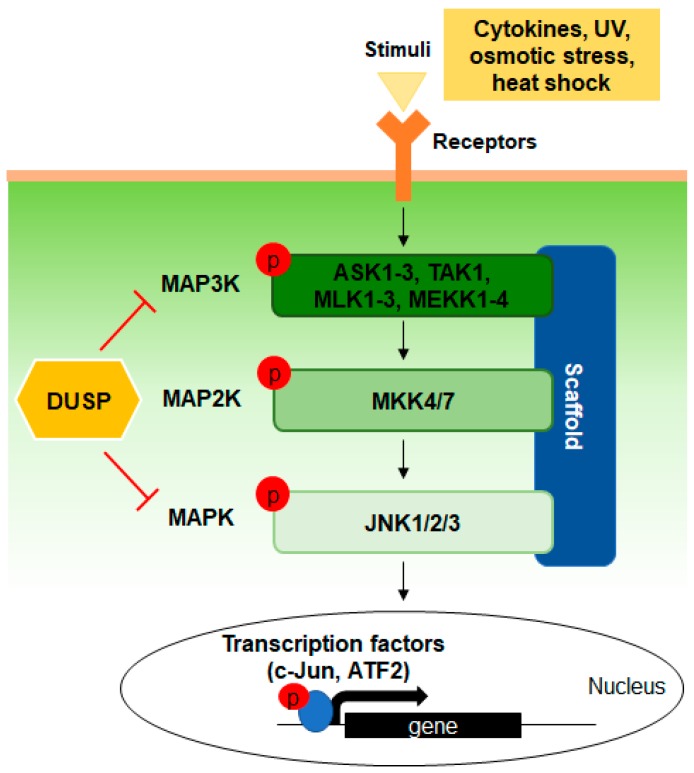
Simplified signal transduction of the c-Jun N-terminal kinase (JNK) pathway. The JNK pathway is activated by extracellular stimuli, including inflammatory cytokines and stress signals. The JNK signaling cascade consists of three kinases: MAP3K, MAP2K, and MAPK, which comprise JNK in this figure. A cascade of kinases forms a signaling complex with scaffold proteins, such as JIP1 or β-arrestin2, which enables efficient signal transduction. When JNK is activated by sequential phosphorylation of upstream kinases, it translocates from the cytoplasm to the nucleus and regulates transcription factors such as c-Jun and ATF2. DUSPs regulate JNK pathway through dephosphorylation of MAP3K and MAPK, while DUSPs dephosphorylating MAP2K have still not been found. JNK, c-Jun N-terminal kinase; MAP3K, mitogen-activated protein kinase kinase kinase; MAP2K, mitogen-activated protein kinase kinase; MAPK, mitogen-activated protein kinase; JIP1, JNK-interacting protein 1; ATF2, activating transcription factor 2; DUSP, dual-specificity phosphatase; UV, ultraviolet; ASK1-3, apoptosis signal-regulating kinase 1-3; TAK1, transforming growth factor beta-activated kinase 1; MLK1-3, mixed-lineage kinase 1-3; MEKK1-4, mitogen-activated protein kinase kinase kinase 1-4; MKK4/7, mitogen-activated protein kinase kinase 4/7.

The JNK pathway is primarily activated by pro-inflammatory cytokines or stress signals, including ultraviolet irradiation, osmotic stress, and heat shock (Figure 3). MAP3Ks of the JNK pathway include apoptosis signal-regulating kinases 1-3 (ASK1-3), transforming growth factor β-activated kinase 1 (TAK1), mitogen-activated protein kinases kinase kinase 1-4 (MEKK1-4), mixed-lineage protein kinase 1-3 (MLK1-3), dual leucine zipper-bearing kinase (DLKs), and leucine zipper-bearing kinases (LZKs). [18,19]. Activation of MAP3Ks leads to phosphorylation and activation of MAP2Ks, mitogen-activated protein kinase kinase (MKK) 4 and MKK7; these proteins then phosphorylate JNK sequentially at threonine and tyrosine residues within the activation loop [20]. The sequential phosphorylation from MAP3Ks to MAP2Ks, then to MAPKs within the JNK pathway is mediated by complex formation with scaffold proteins such as JNK-interacting protein-1 (JIP1) or β-arrestin2, which enables efficient signal transduction [21,22,23]. Although MKK4 and MKK7 phosphorylate JNK, they target different phosphate acceptor sites: MKK4 targets Tyr185 while MKK7 targets Thr183 [24]. The phosphorylation of JNK is estimated to induce a conformational change in its activation loop that creates a functional active site by realigning the N- and C-terminal domains [25]. As activated JNK moves into the nucleus, JNK catalyzes the phosphorylation of a protein substrate by forming a ternary complex with its downstream substrate and transferring the γ-phosphate of ATP. JNK predominately phosphorylates the N-terminal Ser63 and Ser73 residues of c-Jun, a member of activator protein 1 (AP-1) transcription factor family, thus enhancing its transcriptional activity [26,27]. Other downstream substrates of JNK are transcription factors, including members of the activating transcription factor (ATF) family, c-Myc, p53, nuclear factor of activated T-cells-4 (NFAT4), and Elk-1 and non-transcription factors, including the Bcl-2 family [28,29,30,31].

Mammalian genomes contain three closely-related JNK genes: *JNK1* (also known as *MAPK8* or *SAPKγ*), *JNK2* (*MAPK9* or *SAPKα*), and *JNK3* (*MAPK10* or *SAPKβ*) [28,32,33]. Members of the JNK family are divided into two types based on the expression pattern: JNK1 and JNK2 are ubiquitously expressed, and JNK3 shows tissue-specific expression primarily in the brain [34,35]. The JNK family members comprise two different isoforms, α and β, that are formed by alternative splicing [21,25,36,37]. These two JNK isoforms can have different substrate-binding affinities or enzymatic activities. For example, JNK2β-isoforms have lower Michaelis-Menten constants for downstream substrate ATF2 than α-isoforms, which indicates that JNK2β-isoforms have higher binding affinity for ATF2 than α-isoforms [28,38]. However, much remains to be elucidated regarding biochemical and functional differences among JNK isoforms.

Although JNKs share a common structure, they differ in their catalytic activities on substrate proteins; these differences influence diverse biological functions. Dysregulation of the JNK pathway causes uncontrolled activation of downstream substrates, which can eventually lead to disease-like states [21]. Therefore, to regulate the duration and magnitude of JNK activities in response to both physiological and pathological stimuli, phosphatases are essential.

## 3. Phosphatases in Mitogen-Activated Protein Kinase (MAPK) Pathways

Dephosphorylation by phosphatases can be simply understood as reverse phosphorylation. However, considering that kinases of MAPK pathways are inactivated even in the presence of extracellular stimuli, it can be assumed that phosphatases mediate elaborate regulation in terms of both magnitude and duration of signals [39,40]. MAPK signaling cascades have critical features that have made MAPK an attractive pathway for studying signaling dynamics [41]. (1) MAPK is a highly conserved pathway among eukaryotes from yeast to humans, which enables investigations that range in scope from relatively simple forms of the pathway to more complex signaling networks. (2) The pathway is activated by sequential phosphorylation of three tiers of kinases (MAP3K, MAP2K, and MAPK). (3) The kinases form a complex with scaffold proteins that increase the local concentration and enhance the efficiency of signal transduction, a property that allows the study of protein-protein interaction and diffusion effects. (4) Phosphatases inactivate the kinases in MAPK pathway. Therefore, numerous studies have focused on the analysis of MAPK signaling dynamics, both experimentally and theoretically [42,43,44,45]. Notably, phosphatases have been reported to regulate signal transduction within MAPK pathway more dynamically than kinases [42]. Bhalla et al. found that phosphatases controlled the signaling flux of MAPK pathways [42]. In their computational analysis study, phosphatases regulated signal flexibility of MAPK pathways, forming a proportional response system to stimulus as the expression level of phosphatase was increased, which suggests that phosphatases are critical for the flexible signal flow of MAPK pathways [42]. Since MAPK signaling is regulated spatio-temporally, not only catalytic activity but also the expression level and spatial concentration of phosphatases affect the dephosphorylation process [41]. Interestingly, the expression of phosphatases that dephosphorylate MAPK family proteins is often induced by MAPK signaling activities [46,47,48]. Previous studies have also reported that the activity and stability of a phosphatase are regulated by MAPKs [49,50]. Altogether, phosphatases, as well as kinases that participate in MAPK signaling cascades, comprise and regulate complex signaling networks.

## 4. Dual-Specificity Phosphatases (DUSPs): Regulators of the JNK Pathway

**Figure 4:**
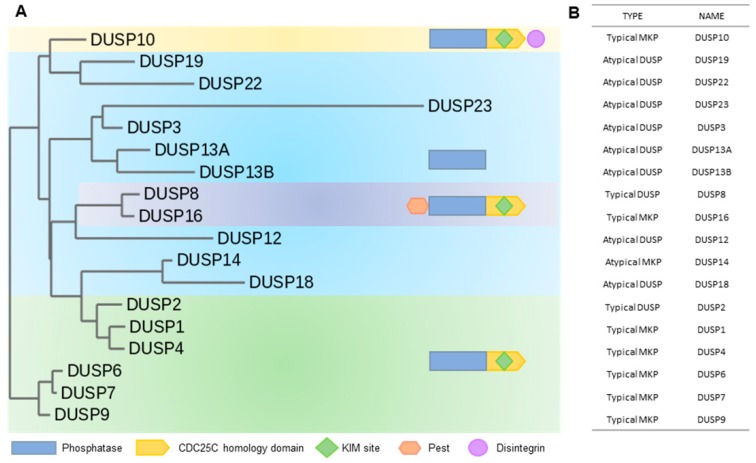
Phylogenetic tree showing the protein sequence similarity of JNK pathway-regulating DUSPs and their classification. (**A**) Based on the protein sequences of the DUSPs that regulate JNK pathway, a phylogenetic tree was constructed. The protein sequences of DUSPs were all obtained through NCBI, and the tree was created using the “One Click” mode provided by Phylogeny.fr (http://www.phylogeny.fr/simple_phylogeny.cgi) [103,104,105,106,107,108,109]. Interestingly, the DUSPs that play similar roles in controlling JNK pathway are located close together. (**B**) DUSPs are listed according to the type and structure analysis of each DUSP. In most cases, proteins with similar domains are not of the same DUSP type, but seem to be somewhat related.

The full human genome sequence predicts more than 500 putative protein kinases, while the predicted number of phosphatases is only ~150 [51,52]. Protein phosphatases have been historically categorized as either protein tyrosine phosphatases (PTPs) or Ser/Thr phosphatases (PPs). In general, PTPs are divided into Class I, including DUSPs and classical PTPs; Class II, consisting of LM-PTP; Class III, comprised of cell division cycle 25 (CDC25) proteins; and Asp-based PTPs [51]. Phosphatases of Class I, II, and III are Cys-based, which indicates that they have a conserved catalytic motif of H/V-C-X-X-X-X-X-R (H/VCX_5_R) containing cysteine, a catalytically active moiety [53]. Because PTPs share a highly conserved catalytic domain containing H/VCX_5_R, the substrate specificity of PTPs arises from a non-catalytic regulatory or interacting domain [53]. However, when comparing DUSPs with the Tyr-specific classical PTPs that belong to Class I, DUSPs have less sequence similarity than classical PTPs, which may suggest a wide range of target substrates and various effects [54]. DUSPs are classified primarily based on the presence of a kinase-interacting motif (KIM). If a DUSP contains a KIM, then it is classified as a typical MAP kinase phosphatase (MKP) or typical DUSP [55]. An atypical DUSP or MKP does not have a KIM [55]. With the type and domain of DUSPs, a phylogenetic tree was constructed in Figure 4. However, this historical classification has recently become less clear-cut, as new DUSPs are discovered and their structures elucidated. That is why the DUSPs of the same type are not clustered together. Various DUSPs are listed in Table 1 and will be discussed in detail in the following sections.

### 4.1. Negative Regulation of JNK by DUSPs

Most DUSPs negatively regulate the JNK signaling pathway via dephosphorylation. As JNK is activated by dual phosphorylation at threonine and tyrosine of the TPY motif, the negative regulation of JNK by DUSPs is typically mediated by dephosphorylation of these residues.

Although nuclear DUSP1 dephosphorylates not only JNK but also ERK and p38, it preferentially binds to JNK [56,57]. Stable expression of DUSP1 inhibited the activation of JNK in COS-7 cells [59]. Suppressing DUSP1 expression causes increased and sustained activation of JNK [58], which indicates that DUSP1 functions as a regulator of both the magnitude and duration of JNK signaling. These results suggest that DUSP1 is essential for regulating the level of p-JNK in the early stages of JNK signaling. Furthermore, superoxide-dependent degradation of DUSP1 in liver cancer cells contributed to the activation of JNK and led to the eventual death of the cancer cells [60]. Interestingly, the induction of DUSP1 was mediated by JNK-downstream transcription factors c-Jun and ATF2 that bound to conserved ATF sites in the *DUSP1* promoter region [61]. As induced DUSP1 dephosphorylates JNK in the nucleus and suppresses further activation of these transcription factors, DUSP1 has a negative feedback effect on JNK signaling.

DUSP2 was initially reported to dephosphorylate only ERK2 in vivo and in vitro, not JNK. [110,111]. However, recent studies show that DUSP2 interact with JNK1, an interaction that is dependent on the KIM of DUSP2, LLRRRAR [62]. Furthermore, DUSP2 is reported to function as a negative regulator of JNK based on an analysis of the JNK pathway in DUSP2^−/−^ mice [62]. As there is a lack of evidence about the negative regulation of JNK by DUSP2, further studies on the function of DUSP2 in JNK signaling pathway are needed.

As a negative regulator of JNK, DUSP3 effectively dephosphorylates JNK2 with a *k_cat_*/*K_M_* of 40,000 (s^−1^ M^−1^) in vitro and downregulates JNK1/2 phosphorylation in response to stress signals in NIH3T3 or COS-1 cells [63,64]. Another study demonstrated that loss of DUSP3 resulted in the hyperactivation of JNK and cell-cycle arrest [65]. Interestingly, JNK that specifically formed a complex with c-Jun were protected from dephosphorylation by DUSP3 in vitro because DUSP3 was suppressed from accessing the phosphorylated sites of JNK [64]. It is assumed that proper binding and orientation of phosphorylated JNK are essential for dephosphorylation by DUSP3.

When mouse embryonic fibroblasts (MEFs) from DUSP4^−/−^ mice were stimulated with anisomycin, the levels of p-JNK were increased relative to those in MEFs from DUSP4^+/+^ mice [66]. Interestingly, JNK increased the catalytic activity of DUSP4 [67]. In addition, the expression level of DUSP4 was significantly higher in malignant tissues than in normal tissues [112]. When cells in which DUSP4 expression was suppressed were treated with H_2_O_2_, the levels of p-JNK1/2 remained higher over six hours than that of control cells in which DUSP4 expression was not suppressed [58].

DUSP6 had been known primarily as a negative regulator of ERK [68,69]. However, the substrate preference of DUSP6 varies according to cell type and physiological conditions [113]. Although purified DUSP6 did not bind to JNK2/3 in vitro phosphatase assays [49], when DUSP6 levels were knocked down, the level of p-JNK increased and downstream substrates of JNK, such as c-Jun, p53, and ataxia telangiectasia mutated (ATM), were activated [70]. When DUSP6 was knocked down using short hairpin RNA (shRNA), the levels of p-JNK and p-c-Jun were significantly increased in ST8814 cells upon stimulation with serum [70]. In DUSP6^−/−^ T cells, TCR-mediated JNK phosphorylation levels were ~1.5 fold those of control cells, which led to increased IL-21 production [72]. The basal p-JNK level in resting DUSP6^−/−^ T cells was also higher compared to control cells, while the duration of JNK phosphorylation was not significantly altered by DUSP6 knockout [72]. In addition, DUSP6 expression reduced JNK phosphorylation by 75% in primary rat neonatal brain cortex astrocytes cells [71]. Taken together, these findings suggest that DUSP6 regulates the magnitude of JNK signals in various cell types.

DUSP8 is a highly specific inactivator of JNK [74,75]. When DUSP8 was co-expressed with JNK3 in COS-7 cells, it inactivated JNK3 [76]. The endogenous DUSP8 bound to JIP1 in the neuronal cell lines ND7 and N1E-115, and ectopically expressed DUSP8 bound to both JIP1 and JIP2 [77]. Specifically, the p-JNK1α1 level decreased to less than 40% of the control level after 5 min of binding with DUSP8, and it decreased to 20% after 30 min in vitro phosphatase assay [78]. The phosphorylation level of JNK2α2 was more sensitive; it dropped to 15% after 5 min. However, these effects were not observed with the C246S mutant of DUSP8 [78]. Interestingly, DUSP8 itself is regulated by JNK [79]. Phosphorylation at Ser515, Thr518, and Ser520 of DUSP8 by JNK results in the attenuation of DUSP8 action. [74]. Altogether, this complex regulation of JNK by DUSP8 (and vice versa) is an excellent example of feedback regulation in signaling networks.

Human DUSP10 inactivated JNK in vitro, an observation that is supported by the finding that JNK activity is enhanced in T cells from DUSP10^−/−^ mice [81,82]. Levels of p-JNK1/2 were significantly increased after two hours of H_2_O_2_ stimulation in DUSP10 knockdown cells compared with the control group [58]. However, after four hours, the level of p-JNK was not different compared with the control, indicating that DUSP10 regulated the early stages of JNK signaling, a pattern that is similar to DUSP1 [58].

DUSP16 inhibited the activation of JNK by dephosphorylating it directly in COS-7 cells [90,91], and the catalytic domain of DUSP16 was responsible for both JNK-binding and enzymatic specificity [90]. Willoughby et al. suggested that DUSP16 binds to JIP-1 and reduces p-JNK and p-c-Jun [77]. ASK1 overexpression causes DUSP16 to dephosphorylate JNK3 that is bound to β-arrestin2 [94]. Furthermore, the regulation of DUSP16 by other MAPKs is an excellent example of signaling pathway networks. When Ser446 of DUSP16 is phosphorylated by ERK, DUSP16 is stabilized by reduced ubiquitination, which results in the further inhibition of JNK [92,93]. Such regulation of DUSP16 in the JNK pathway by ERK exerts crosstalk between pathways, forming an orchestrated signaling network.

DUSP7, DUSP12, DUSP13B, and DUSP18 have also been analyzed as dephosphorylating and inactivating regulators of JNK [73,83,95,114]. When DUSP12 was overexpressed in RAW264.7 cells that were stimulated with LPS, the level of p-JNK was significantly reduced over time compared with control cells [114].

Until recently, most studies of DUSPs targeting JNK did not consider the kinetics of JNK regulation, including the magnitude, duration, and frequency of JNK signal flow. Nevertheless, as more DUSPs have been identified as essential JNK pathway regulators, DUSPs have become attractive targets for understanding the JNK pathway from diverse perspectives.

### 4.2. DUSPs Acting on other JNK Signaling Kinases

Although most DUSPs have been found to negatively regulate JNK as discussed in Section 4.1, novel functions with other upstream kinases of the JNK pathway are being investigated.

The activities of ASK1 and its downstream target MKK4/7 are induced by DUSP9 deficiency and inhibited by DUSP9 overexpression in hepatic steatosis, indicating that DUSP9 regulates the JNK pathway and related metabolic disorders by dephosphorylating ASK1 [80]. DUSP12 also attenuated JNK signaling by directly binding to and dephosphorylating ASK1 in the human normal hepatocyte cell line L02 [115]. Huang et al. also suggested that DUSP12 suppresses ASK1 activity and hepatic lipotoxicity induced by the oxidative and ER stress of a high-fat diet [115]. DUSP14 not only inactivates JNK in vitro [85] but also dephosphorylates TAK1-binding protein 1 (TAB1), which leads to TAK1 inactivation [93,94,95]. Because TAK1 is an upstream regulator of JNK1/2, the downstream of the JNK signaling cascade is inactivated. Another study suggested that activated DUSP14 directly interacted with TAK1 [89].

Several DUSPs regulate the JNK pathway by directly or indirectly dephosphorylating upstream kinases as well as JNK. So far, we have discussed DUSPs that downregulate the JNK pathway, but some DUSPs induce the upregulation of JNK signaling.

### 4.3. DUSPs as Scaffolds of JNK Signaling

DUSPs have also been shown to function as scaffold proteins that form complexes with kinases of the JNK pathway.

One study suggested that DUSP13A induced ASK1 to induce apoptosis by activating caspase-3 through attachment to the N-terminal portion of ASK1 [84]. The level of p-JNK was increased by co-expression of ASK1 and DUSP13A but not by the independent expression of ASK1 or DUSP13A [84]. The same results were observed when a catalytically inactive mutant of DUSP13A was ectopically expressed [84]. Because the regulation of ASK1 by DUSP13Awas independent of its phosphatase activity, DUSP13A might function as a scaffold protein that binds to ASK1 to activate the JNK pathway [84].

DUSP19 was reported to bind directly to MKK7 and reduce JNK activity when expressed in COS-7 cells [96]. Zama et al. demonstrated that DUSP19 functioned as a scaffold of the JNK pathway [97]. ATF2 was suppressed by a relatively low expression of DUSP19, while the signaling flux was increased by higher levels of DUSP19 expression [97]. The inhibition of JNK signaling by reduced expression of DUSP19 was independent of the catalytic domain of DUSP19, indicating a scaffold-like role for DUSP19 [97].

An early study suggested that DUSP22 could dephosphorylate and inactivate JNK signaling in COS-7 cells [98]. However, other studies showed that DUSP22 activated JNK [99]. The level of p-JNK was increased via the enhanced activities of MKK4/7 when JNK and DUSP22 were co-expressed in COS-7 or HEK293 cells [99,100]. DUSP22 was required for the full activation of JNK in mouse embryonic stem cells [100]. A recent study revealed that DUSP22 functions as a scaffold protein that binds to ASK1, MKK7, and JNK to regulate apoptosis independent of its phosphatase activity [101]. The enhancement of the JNK pathway by DUSP22 shown in other previous studies may be the result of DUSP22 playing a scaffold-like role.

Increased phosphorylation of MKK4 and MKK6 and enhanced activity of JNK and p38 were observed with DUSP23 expression in COS-7 cells [102]. Because of these data, Takagaki et al. suggested that DUSP23 plays a scaffold-like role in the activation of JNK [102].

Although DUSPs acting as scaffolds differ in their respective activation and inactivation mechanisms for the JNK pathway, it is evident that they accelerate the process and function as positive regulators of JNK signaling. As these DUSPs regulate JNK-induced intracellular signal transduction via their phosphatase activities and/or scaffold-like binding affinities, a therapeutic strategy targeting DUSPs is expected to spur the development of novel therapeutic strategies.

## 5. Effects of DUSPs in JNK-Associated Diseases

The JNK pathway is involved in a multitude of diseases ranging from cancer to dysfunctions of the immune and nervous systems. Studies show that a high level of JNK activity is detected in some cancer cell lines [116,117]. As described in Section 4.1, Section 4.2 and Section 4.3, most DUSPs function as negative regulators and some as positive regulators within the JNK signaling cascade. Therefore, a defect in DUSP activities has potential to cause dysfunction of the JNK pathway.

A study by Wu and Bennette showed that the loss of DUSP1 caused an increase in stress-induced JNK activity in MEFs and reduced cell growth [118]. Another study with DUSP1-deficient mice showed a failure in downregulating stress-induced JNK signaling in immune cells such as macrophages and dendritic cells [119]. This study revealed that the loss of DUSP1 led to an enhanced JNK activity and eventually increased the expression of pro-inflammatory cytokines, such as IL-6, IL-12, TNF-α, and IFN-γ, in macrophages exposed to LPS [119,120]. In addition, overexpression of DUSP1 resulted in a neuroprotection effect in rat model of Huntington’s disease by inhibiting apoptosis in primary striatal neurons [121].

DUSP4 was found to be epigenetically silenced by aberrant DNA methylation in a majority of diffuse large B-cell lymphomas [122]. The deficiency of DUSP4 contributed to cancer progression by increasing JNK activity [123]. It was consistent with the results that ectopic expression of DUSP4 mediated JNK suppression and induced apoptosis in B-cell lymphoma cell lines [123].

DUSP16 is silenced due to methylation in Burkitt’s lymphoma (BL); therefore, JNK signaling is deregulated in these cells [124]. The ectopic expression of DUSP16 resulted in delayed and suppressed JNK phosphorylation [124], which showed that restoring DUSP16 attenuated irregular JNK activation. Therefore, to regulate JNK signaling in cancer cells such as BL [124], DUSPs that function as negative regulators of the JNK pathway are needed.

Because JNK signaling plays different roles according to cell type and extracellular stimuli, it can appear to perform very different functions. For example, the dysfunction of JNK may lead to uncontrolled proliferation or enhanced migration that is often found in cancer, while dysregulation of JNK signaling may stimulate apoptosis in neuron systems. Therefore, DUSPs that affect the JNK pathway need to be studied under various conditions and with multiple disease models to expand our understanding of their regulatory mechanisms.

## 6. Possibilities for DUSP inhibitors

Therapeutic approaches that involve phosphatase inhibitors have lagged behind those that involve kinase inhibitors. However, based on increasing evidence and elucidation of phosphatase mechanisms, novel candidates for inhibiting phosphatases are being investigated to address diseases in which JNK signaling is abnormal (Table 2).

Arsenite induced the activity of JNK and directly inactivated DUSP8 in HEK293 cells that stably express DUSP8 [79]. Arsenite induced phosphorylation at Ser515, Thr518, and Ser520 of DUSP8 in HEK293T cells, which attenuated DUSP8 activity [74]. Rosiglitazone is an agonist for synthetic peroxisome proliferator-activated receptor-γ (PPAR-γ), and several studies have shown that rosiglitazone elicited neuroprotective effects in animal models of brain disease [125,126,127]. The protective effect of rosiglitazone that attenuated cell death after ischemia was mediated by upregulated DUSP8 activity that blocked ischemia-induced phosphorylation of JNK [125]. PTP inhibitor IV was confirmed to inhibit DUSP14-mediated dephosphorylation of JNK in vitro and in vivo [128]. In addition to suppressing DUSP14, NSC95397 is also known to inhibit DUSP1 and 6 [129,130]. Other chemicals that inhibit DUSP1 and 6 include sanguinarine [131], adociaquinone B, naphthoquinone derivatives [132], TPI-3 [133], and BCI [134]. TPI-3 was found from chemical databases by computer-assisted structure analyses with TP1-2, and selectively increased p-JNK in Jurkat cells in vitro [133]. In addition, TPI-2 was identified as a DUSP1 inhibitor by chemical screening [133]. BCI was predicted to preferably bind to the gap between helical α7 and the general acid loop of DUSP6 rather than directly binding catalytic residues of DUSP6 through computational modeling prediction method [134]. In addition, the target inhibition of DUSP1 by BCI induced JNK activation in highly aggressive malignant peripheral nerve sheath tumors, which diminished cell survival in vitro and caused tumor necrosis in vivo [70]. RK-682 is a protein tyrosine phosphatase inhibitor that is also known as an inhibitor of DUSP3 [135,136]. AS077234-4 is known as a novel and only one inhibitor of DUSP10 up to date [137]. DUSP22 functions as a scaffold protein of the JNK signaling pathway, and is inhibited explicitly by quinoxalinylurea-based small molecule compound [138]. In addition, rhodamine-based inhibitors selectively inhibited DUSP22 [139].

Although these inhibitors of DUSPs have not yet proceeded into clinical trials, the strategy of using DUSP inhibitors as therapeutics is under active investigation. Novel therapeutic approaches targeting these phosphatases are emerging as studies continue to focus on the mechanisms and regulatory functions of DUSPs.

## 7. Conclusions

DUSPs have a broad spectrum of targets and mechanisms for dephosphorylating serine, threonine, and tyrosine residues that are phosphorylated in ~30% of cellular proteins. Dephosphorylation of kinases comprising the JNK pathway that regulates apoptosis, inflammation, development, and neuronal function is an essential reaction that maintains normal cellular physiology. Because DUSPs are critical regulators of JNK signaling pathways, approaches targeting DUSPs would spur the development of novel therapies for JNK-related diseases.

## Figures and Tables

**Table 1:** Dual-specificity phosphatases (DUSPs) and their effects in the c-Jun N-terminal Kinase (JNK) pathway.

Name	Alternative Names	Target Signaling	Cellular Effects Related to the JNK Pathway	Ref.
**DUSP1**	MKP1, CL100, VH1, PTPN10, HVH1	JNK, p38 > ERK1/2	■ Inhibits the activation of JNK in COS-7 cells ■ Leads cancer cell death in liver cancer cell line ■ c-Jun and ATF2 mediate DUSP1	[56,57,58,59,60,61]
**DUSP2**	PAC-1	ERK1/2, p38 > JNK1	■ Functions as a negative regulator of JNK in DUSP2^−/−^ mice ■ Interacts with JNK1	[62]
**DUSP3**	VHR	JNK	■ Functions as a negative regulator of JNK ■ DUSP3 C124S, catalytically inactive mutant, acts as a substrate trap in vivo ■ Phosphorylation sites of DUSP3 are specifically blocked from c-Jun complexed with JNK in vitro ■ Deletion of DUSP3 leads the cell cycle arrest	[63,64,65]
**DUSP4**	MKP2, VH2, VHV2, TYP	JNK, ERK1/2 > p38	■ Affects the cellular proliferation in embryonic fibroblasts from KO mice	[66,67]
**DUSP6**	MKP3, PYST1	ERK1/2, ERK5 > JNK	■ Functions as a negative regulator of ERK and interacts with ERK2 ■ Could not bind to JNK2/3 in vitro ■ Increases the level of TCR-mediated p-JNK when DUSP6 is suppressed ■ Reduces the level of p-JNK in primary rat neonatal brain cortex astrocytes cells	[49,68,69,70,71,72]
**DUSP7**	MKP-X, PYST2	ERK1/2 > JNK1/2	■ Binds to JNK and leads inactivation	[73]
**DUSP8**	VH5, HVH8, HVH-5 (M3/6 in mouse)	JNK3 > ERK, p38	■ A highly specific inactivator of JNK ■ Inactivates JNK3 when expressed in COS-7 cells ■ Binds to JIP1 in ND7 and N1E-115 cells ■ Regulated by JNK	[74,75,76,77,78,79]
**DUSP9**	MKP4	ERK > p38 > JNK	■ Dephosphorylates ASK1	[80]
**DUSP10**	MKP5	JNK, p38 > ERK	■ Inactivates JNK in vitro ■ Enhances JNK when deleted in T- cells	[81,82]
**DUSP12**	YVH1	JNK	■ Binds directly to ASK1 and dephosphorylates in L02 cells ■ Dephosphorylates and inactivates JNK	[81,82]
**DUSP13**	DUSP13A, DUSP13B, BEDP, MOSP, SKRP4, TMDP	JNK, p38 > ERK	■ DUSP13B dephosphorylates JNK ■ DUSP13A acts as a scaffold protein that binds to ASK1 to activate JNK	[83,84]
**DUSP14**	MKP6, MKP-L	JNK > ERK > p38	■ Inactivates JNK in vitro ■ Dephosphorylates TAB1 ■ Directly interacts with TAK1	[85,86,87,88,89]
**DUSP16**	MKP7	JNK3, p38 > ERK	■ Dephosphorylates JNK directly in COS-7 cells ■ Phosphorylated by ERK ■ Binds to JIP-1	[77,90,91,92,93,94]
**DUSP18**	DUSP20, LNW-DSP20	JNK	■ Dephosphorylates and inactivates the pathway of JNK signaling	[95]
**DUSP19**	SKRP1, DUSP17, LMW-DSP3, TS-DSP1	JNK	■ Binds directly to MKK7 in COS-7 cells ■ Functions as a scaffold of JNK pathways ■ Increases ATF-2 dependent on the expression level of DUSP19	[96,97]
**DUSP22**	JSP-1, JKAPVHX, LMW-DSP2, MKPX	JNK	■ Activates MKK4 in COS-7 and MKK7 in HEK293 cells ■ Dephosphorylates and inactivates JNK signaling in COS-7 cells ■ Binds to ASK1, MKK7, and JNK to function as a scaffold protein	[98,99,100,101]
**DUSP23**	DUSP25, VHZ, LDP-3, MOSP	JNK, p38	■ Induces MKK4 and 6 activations in COS-7 cells	[102]

**Table 2:** DUSP inhibitors.

Name	Target Inhibition DUSPs	Reference
**Arsenite**	DUSP8	[74,79]
**Rosiglitazone**	DUSP8	[125,126,127]
**PTP inhibitor IV**	DUSP14	[128]
**NSC95397**	DUSP1, DUSP6, DUSP14	[129,130]
**Sanguinarine**	DUSP1, DUSP6	[131]
**Adociaquinone B & Naphthoquinone**	DUSP1, DUSP6, CDC25B	[132]
**TPI-3**	DUSP1	[133]
**BCI**	DUSP1, DUSP6	[70,134]
**RK-682**	DUSP3	[135,136]
**AS077234-4**	DUSP10	[137]
**Quinoxalinylurea**	DUSP22	[138]
**Rhodanine**	DUSP22	[139]
